# Repeated Application of Transcranial Diagnostic Ultrasound Towards the Visual Cortex Induced Illusory Visual Percepts in Healthy Participants

**DOI:** 10.3389/fnhum.2020.00066

**Published:** 2020-03-03

**Authors:** Nels Schimek, Zeb Burke-Conte, Justin Abernethy, Maren Schimek, Celeste Burke-Conte, Michael Bobola, Andrea Stocco, Pierre D. Mourad

**Affiliations:** ^1^Department of Neurological Surgery, University of Washington, Seattle, WA, United States; ^2^Department of Psychology, University of Washington, Seattle, WA, United States; ^3^Division of Engineering and Mathematics, University of Washington, Seattle, WA, United States

**Keywords:** diagnostic ultrasound, ultrasound stimulation, visual stimulation, increased sensitivity, transcranial magnetic stimulation

## Abstract

Transcranial magnetic stimulation (TMS) of the visual cortex can induce phosphenes as participants look at a visual target. So can non-diagnostic ultrasound (nDU), delivered in a transcranial fashion, while participants have closed their eyes during stimulation. Here, we sought to determine if DU, aimed at the visual cortex, could alter the perception of a visual target. We applied a randomized series of actual or sham DU, transcranially and towards the visual cortex of healthy participants while they stared at a visual target (a white crosshair on a light-blue background), with the ultrasound device placed where TMS elicited phosphenes. These participants observed percepts seven out of ten times, which consisted of extra or extensions of lines relative to the original crosshair, and additional colors, an average of 53.7 ± 2.6% of the time over the course of the experiment. Seven out of ten different participants exposed to *sham-only* DU observed comparable percepts, but only an average of 36.3 ± 1.9% of the time, a statistically significant difference (*p* < 0.00001). Moreover, on average, participants exposed to a combination of sham and actual ultrasound reported a net increase of 47.9 percentage points in the likelihood that they would report a percept by the end of the experiment. Our results are consistent with the hypothesis that a random combination of sham-only and actual DU, applied directly over the visual cortex of participants, increased the likelihood that they would observe visual effects, but not the type of effects, with that likelihood increasing over the course of the experiment. From this, we conclude that repeated exposures by DU may make the visual cortex more responsive to stimulation of their visual cortex by the visual target itself. Future studies should identify the biophysical mechanism(s) and neural pathways by which DU, in our hands and others, can generate its observed effects on brain function. These observations, consistent with other’s observation of effects of DU stimulation of the human motor cortex and amygdala, as well as the FDA approved nature of DU, may lead to increased use of DU as a means of altering brain function.

## Introduction

Ultrasound can modulate brain function in a targeted and non-invasive way. Much of this work has used non-diagnostic ultrasound (nDU) which differs from diagnostic ultrasound (DU) due to the former’s use of lower frequencies (typically 250–650 kHz) and longer individual pulses (typically hundreds of cycles) than DU (typically greater than 2 MHz and equal to or shorter than three cycles of ultrasound, respectively). Early work with transcranially delivered non-diagnostic ultrasound (nDU) temporarily inhibited the function of the visual cortex of anesthetized cats (Fry et al., 1955) and activated thalamus and other targets within the brains of cats and rabbits (Velling and Shklyaruk, 1988). Tyler and colleagues re-started studies of modulation of brain function with ultrasound in their first articles (Tyler et al., 2008; Tufail et al., 2010), which showed generation by nDU of neuronal activity within hippocampal brain slices from mice. Other, nDU-based studies with rodents followed, demonstrating, for example, motor and eye activity caused by ultrasound stimulation along with a better understanding of the parameters necessary for successful neuromodulation (Tufail et al., 2010; King et al., 2013; Younan et al., 2013; Kim et al., 2014; Ye et al., 2016). Other studies with mice, rats, and rabbits increased our understanding of the anatomical specificity and other biophysical aspects of ultrasound modulation of brain function (Yoo et al., 2011a, 2018; Kim et al., 2012, 2014; Mehić et al., 2014; Kamimura et al., 2016; Airan et al., 2017). Modulation of brain activity in larger animals with nDU has also succeeded, specifically in sheep and non-human primates (Deffieux et al., 2013; Lee et al., 2016b; Wattiez et al., 2017).

In addition to simple transient stimulation effects, nDU has shown potential for therapeutic applications. For example, stimulation of the thalamus of anesthetized rats can reduce the time required for them to recover from the anesthesia (Yoo et al., 2011b), while thalamic stimulation of a recently comatose patient produced significant clinical improvement (Monti et al., 2016). Our group has successfully applied nDU to a mouse model of multiple sclerosis, thereby demonstrating accelerated remyelination in mice (Olmstead et al., 2018); another group showed a reduction of acute epileptic seizure activity in rats (Min et al., 2011). Also, nDU application to the thalamus of swine inhibited down-stream sensory evoked potentials (Dallapiazza et al., 2018) with a variety of potential uses.

Recent studies have applied nDU to humans. For example, nDU applied to the somatosensory cortex has generated increased sensory discrimination while attenuating sensory evoked potentials (Legon et al., 2014), modulated electroencephalographic (EEG) dynamics (Mueller et al., 2014), and generated tactile sensations (Lee et al., 2015). Also, when applied to the visual cortex of healthy participants with their eyes closed, nDU activated the target cortical area as demonstrated with EEG and the induction of phosphenes (Lee et al., 2016a).

Finally, there exist two studies known to us that report temporary changes in brain function using transcranially delivered DU (DU), each applied to humans. In one study, researchers applied DU through the temporal window towards the amygdala, thereby producing improved self-reported mood in patients with chronic pain (Hameroff et al., 2013). Another study (Gibson et al., 2018) applied DU directly over the motor cortex of healthy participants, observing an increased likelihood that subsequent application of transcranial magnetic stimulation (TMS) to the same motor cortex would generate motor evoked potentials.

Motivated by observations of alterations of brain function discernible by the participants themselves (Hameroff et al., 2013; Legon et al., 2014; Mueller et al., 2014; Lee et al., 2015, 2016a; Losey et al., 2016; Gibson et al., 2018), including by DU, we sought as our primary goal to determine if DU could alter the visual perception of a target (that is, their “percept”) when applied at the anatomical location where TMS could induce phosphenes. As a secondary goal, we sought to determine if the hypothesized effects on the visual cortex arose due to the short-term or long-term effects of ultrasound exposure.

## Materials and Methods

### Participants

Twenty-one healthy participants (age range 18–44 years, mean 21 years) took part in the experiment in exchange for modest monetary compensation. All participants gave informed consent prior to the experiment. After signing a consent form each subject completed a TMS inclusion criteria screening addressing major head injuries, seizures, stroke, epilepsy, color blindness or any additional health-related conditions that, coupled with TMS exposure, could pose a risk to their health. Participants were not immediately excluded if any of these conditions were present, but a discussion commenced as to whether their condition was severe enough to pose a risk to their health or to impact the experiment. None of the twenty-one participants were excluded based on these screening criteria. The experimental protocol was approved by the University of Washington’s Institutional Review Board (IRB Application #48643).

### Overview of the Experiment

**Figure 1:**
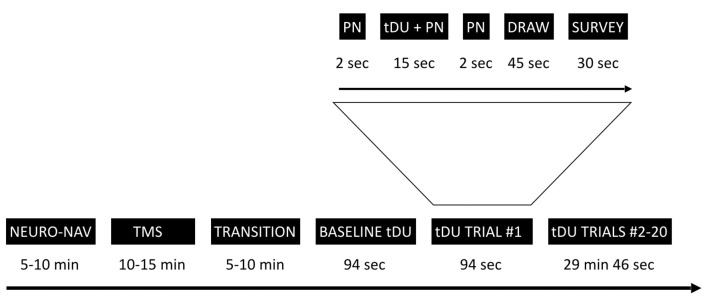
Experimental timeline. The study starts with the neuro-navigation step (NEURO_NAV), which places the participant and devices in the same coordinate system—Figure 2. The transcranial magnetic stimulation step (TMS) identifies a place in the visual cortex amenable to external stimulation, at least by TMS—Figure 3. We then move to transcranial diagnostic ultrasound (DU) after a time of transition. After gelling the participant’s hair (Figure 4) and setting up the DU system in the neuro-navigation setup (Figure 5) we perform a baseline DU step to allow the participant to define for themselves what they see without any DU stimulation. Following this is 20 trials of sham or actual DU exposure, each with the following format: 2 s of increasing pink noise (PN), followed by 15 s of sham or actual DU plus constant pink noise (DU + PN), followed by 2 s of descending pink noise. Participants then have 45 s to draw what they observed and 30 s to answer a survey question, regardless of whether or not they observed a percept.

Figure 1 provides an overview of the experimental timeline. Briefly, we first used a commercial neuro-navigation system to locate the visual cortex of a given participant, then TMS to their visual cortex to determine whether or not a given participant perceived phosphenes when exposed to TMS stimulation. For those participants who did, we then exposed or sham-exposed them to DU applied to the center of the same spot activated by TMS using a single-blinded protocol. Through a series of questions after each ultrasound exposure, we elicited the participant’s self-report of what they experienced. We note here that during each of TMS and sham/actual ultrasound exposure, we turned off the lights in the room, thereby allowing the participant to focus on the computer screen in front of them.

The preceding material effectively summarizes our instructions to the participants, which we have appended to this article as Supplementary Material.

### TMS Localization

**Figure 2:**
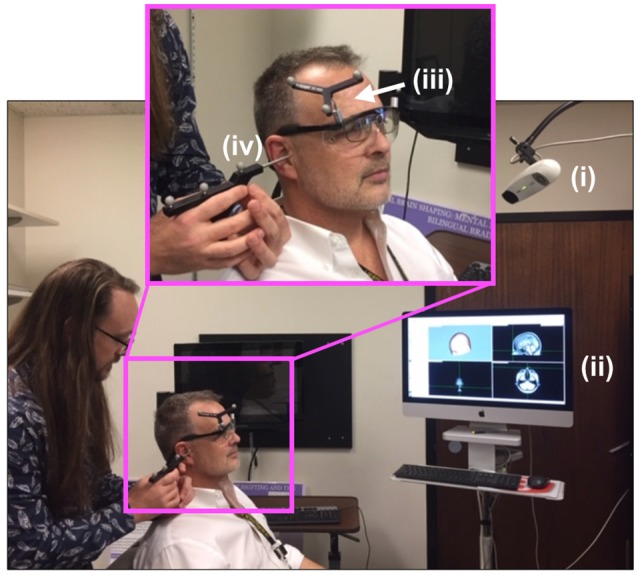
Neuro-navigation setup. To define a coordinate system for subsequent placement of the TMS and DU devices, the (i) infrared navigation system facilitated projection of (ii) a standard head model onto the participant’s head (PDM here), identified grossly by (iii) an infrared-reflective marker placed at a predetermined position on the participant’s head then in a more refined way through placement (by JA here) of the tip of a (iv) pointing device (the “phaser”), with infrared reflective markers on each ear as shown, and the bridge of the nose.

Figure 2 shows the experimental setup for the first, neuro-navigation step. A stereotactic neuro-navigation positioning system (BrainVision BrainSight, Montreal, Canada, with documented submillimeter precision) based on an infrared camera (Polaris) was used to track in real-time the absolute position of the participant’s head and the TMS coil using reflective markers placed on both the coil and the participant’s head. An approximate neuroanatomical reference was obtained by aligning each participant’s head with a standard magnetic resonance imaging (MRI) model (ICBM152 from the Montreal Neurological Institute); the alignment process was based on minimizing the squared distance between known anatomical landmarks (the nasium, left and right ear) on each participant and on the template. A generic anatomical template was then aligned using these measurements to create a model of the scalp and underlying brain areas. A location one cm superior and one cm left of the inion. was identified and selected as the target for TMS stimulation; this location typically corresponds to the most exposed part of an individual’s scalp and has been used multiple times in our laboratory (Stocco et al., 2015; Jiang et al., 2019).

**Figure 3:**
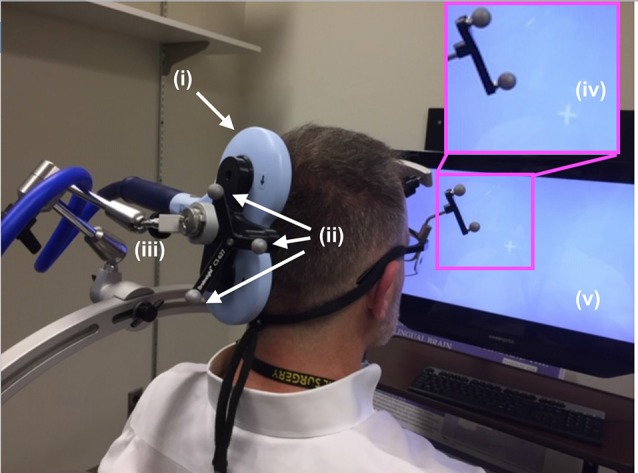
TMS setup. The (i) TMS coil, registered into the infrared navigation system thanks to its (ii) infrared reflective markers was then deployed on an (iii) articulated arm to facilitate refined movement of the coil as needed while the participant experienced phosphenes in the periphery of their view of the (iv) white visual target projected on the (v) computer screen. As shown in Figure 5, participants rested their chin and forehead on a device during the entirety of the TMS procedure.

Once this anatomical location was found, the center of the TMS Figure-8 coil, as indicated in the neuro-navigation system, was placed over this location using a clamp and articulating arm that held the TMS coil—Magstim^®^ Rapid^2^ TMS system (Magstim Inc., Whitland, UK)—Figure 3.

During phosphene thresholding, subjects were asked to fixate on a crosshair in the center of a computer monitor positioned in front of them (Figure 3, inset). They were instructed to be vigilant to the presence of any visual changes during TMS stimulation and to report the presence or absence of phosphenes (by definition the percept generated by TMS) after each stimulation. Participants received 3–5 single TMS pulses at a relatively low-intensity level, starting with the initial targeted area described above. Subjects were asked to report on the presence or absence of a phosphene. If no phosphene was detected, the TMS stimulation intensity was increased by 5%. This procedure continued until the participant reported the presence of phosphenes during stimulation or a stimulation intensity of 85% of the maximum was reached. In the absence of phosphenes at this initial target, locations 0.5–1 cm from this location in the left and upwards directions were then evaluated using the same ramp-up procedures. The position that elicited clear and consistent phosphenes was recorded in the neuro-navigation system and then used to position the application of ultrasound stimulation using the same neuro-navigation system.

### Ultrasound Exposure Protocol

Setup for the ultrasound portion of the experiment began immediately after completion of the TMS localization portion of the study, taking approximately 5–10 min to complete. For our study, we used an L25x 13–6 MHz transducer attached to a Sonosite M-Turbo Ultrasound system (FUJI Sonosite, Bothell, WA, USA). We set the device to “penetration mode” and its gain to its highest setting, in order to maximize the amount of ultrasound to cross the skull. The Mechanical Index (MI) of the device was 0.7 during stimulation. When we applied ultrasound we did so by releasing the “freeze” function of the device and stopped by turning the “freeze” function back on, a single-blinded approach used in other studies involving DU (Conover and Iman, 1979; Bailey et al., 2016).

**Figure 4:**
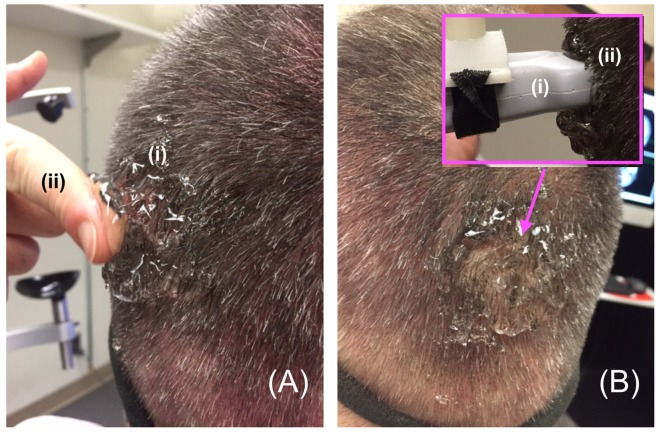
Gelling procedure. **(A)** For participants responsive to TMS stimulation, we placed a copious amount of (i) gel on and around the scalp at the point of projection of the centroid of the TMS device then used a (ii) finger to **(B)** thoroughly rub the gel into the participant’s scalp, before placing more gel on the same spot and then putting the distal tip the (i) ultrasound transducer on that (ii) spot—inset.

**Figure 5:**
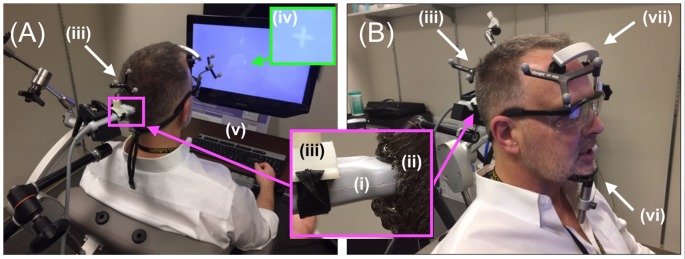
Diagnostic ultrasound setup. (**A,B**, inset) Through the use of the neuro-navigation system, investigators placed the distal tip of the (i) ultrasound scan head into the (ii) hair of the participant at the projection of the centroid of the TMS coil, aided in that placement by strategically placed (iii) infrared reflective markers. Participants observed (iv) white visual target projected on the computer screen with access to (v) a keyboard. **(B)** Participants rested their (vi) chin and (vii) forehead on a device during the entirety of the ultrasound (and TMS) procedure.

In order to facilitate maximum propagation of ultrasound from the device into the participant’s visual cortex while they viewed the visual target, we placed a copious amount of Aquasonic Clear^®^ ultrasound gel at the point of successful TMS stimulation (Figure 4A) that we rubbed throughout the hair down to the scalp (Figure 4B). We then connected the ultrasound transducer to the 3D tracking system and scan-head holder to facilitate precise placement of the ultrasound transducer within the gel on the scalp over the location of the centroid of the TMS probe that allowed successful TMS stimulation (Figure 5).

Participants were given headphones as well as a keyboard placed within easy reach of the participant in a way that allowed them to view the screen and its visual target (Figure 5A). The keyboard allowed the participant to signal when they first observed a visual effect and when that effect ended. It was connected to a laptop with a stopwatch that started when the participant pressed the spacebar and stopped when they pressed it again. The headphones allowed us to use sound to mask the hum of the DU transducer, audible to some participants. Specifically, at the start of each trial, pink noise (PN) played in the participant’s headphones for 19 s, with the volume increasing during the initial 2 s and decreasing over the final 2 s (Figure 1, inset). Seconds 3–17 of the total 19 s contained a stable volume of noise and overlapped in time with the 15-s window for the stimulation phase.

### The Temporal Pattern of Ultrasound Exposure

We incorporated two control exposures in our study, one within and one between subjects. Eleven participants underwent 21 total consecutive trials with exposure to a random distribution of sham (the first control exposure, within a participant, and different for each participant) or actual ultrasound exposures. Ten separate participants underwent 21 total consecutive trials with only sham ultrasound applied throughout the study (the second control exposure, between cohorts). After exposure to sham or actual ultrasound for 15 s interleaved with pink noise as described above, there followed a drawing phase (45 s), during which the participant drew what they observed. After the drawing phase, participants answered the following survey questions projected onto the computer screen for an additional 30 s:

1. Relative to the crosshair, was the percept brighter or dimmer when it began?
2. Did the brightness of the percept change over time?
3. Did elements of the percept change in thickness?
4. Did elements of the percept shorten or lengthen?
5. Did the percept move independently of your gaze?

Each phase lasted for the same duration regardless of whether or not the participant experienced a percept, thereby ensuring each participant experienced the same duration of the study and that stimulation phases were temporally equidistant from one another. All trials used the same visual target: same white crosshair on a light gray background projected onto a computer screen placed approximately 50 cm in front of the participant.

The first trial—Trial 1—defined the baseline visual experience for each participant. As such it always did not involve the actual application of ultrasound as explained to the participant before the start of the study. Participants noted their visual experiences during the baseline trial and were asked to report during subsequent trials any changes of their visual experience relative to this baseline trial. During the 20 subsequent trials, participants were not told whether or not actual or sham ultrasound was applied. For the 11 participants exposed to a combination of sham and actual ultrasound, six of the subsequent 20 trials were assigned as sham ultrasound trials, whose distribution was determined using a random number generator, with unique distributions for each participant. The remaining 14 trials involved actual ultrasound exposure. The separate control group of ten different participants received only sham ultrasound exposure.

### Data Analysis

#### Subject Response Rate Sliding Window Analysis

**Figure 6:**
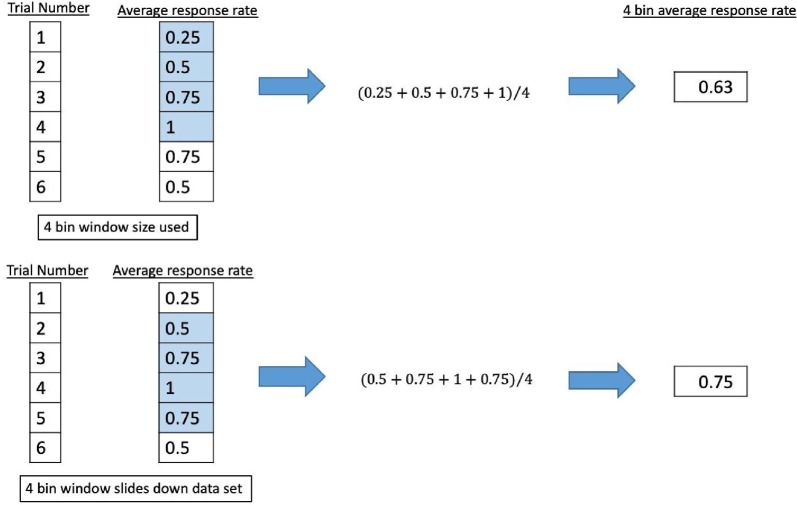
Example of sliding window analysis using a 4 bin window.

A binary coding system was applied to the participant response of observed percepts at each trial (Fry et al., 1955; Velling and Shklyaruk, 1988; Tyler et al., 2008; Tufail et al., 2010; Min et al., 2011; Yoo et al., 2011a,b, 2018; Kim et al., 2012, 2014; Deffieux et al., 2013; King et al., 2013; Younan et al., 2013; Mehić et al., 2014; Kamimura et al., 2016; Lee et al., 2016b; Monti et al., 2016; Ye et al., 2016; Airan et al., 2017; Wattiez et al., 2017; Olmstead et al., 2018), using “1” to signify a reported percept report and “0” for none. The data series was smoothed by passing it through different sliding windows where the average response rate was calculated for bins of four, five, eight, or 10 consecutive trials. The window (keeping the same length of trials) would move down one trial number and the average response rate for that window would be calculated until it reached the end of the data set. We show an example in Figure 6.

#### Statistical Analysis

We first applied the Kruskal–Wallis equality-of-populations rank test (a non-parametric equivalent to ANOVA) to determine if there exists a significant difference between reported experiences of the sham-only cohort vs. the sham plus actual ultrasound cohort, across all bin values. After achieving a positive result with Kruskal–Wallis, we then applied the two-sample Wilcoxon rank-sum (Mann–Whitney) test, a non-parametric equivalent to *t*-test paired Student’s *t*-test, to the average across all trials of each windowed data set. Together, this two-step analysis allows us to compare the time-averaged responses of the sham-only cohort with the sham plus actual ultrasound cohort across all trials without risk of false positives arising from unaccounted for multiple comparisons (Conover and Iman, 1979).

To develop a deeper understanding of the results, including an understanding of the temporal evolution of the participant responses, we then applied a generalized estimating equation (GEE) model to the raw un-windowed data associated with those participants who reported percepts from the sham plus actual ultrasound cohort, excluding baseline measurements. This approach is, in essence, a nonlinear regression analysis of the data (Hin et al., 2007). The model takes as its input data the trial type (sham or actual ultrasound exposure) and the response of the participant in a given trial (whether or not they reported a percept).

Our GEE model starts with the application of an autoregression correlation term (Hin et al., 2007). This mathematical expression accounts for possible contributions of the actual reporting of percepts, not trial type, observed during previous trials on the next sham or ultrasound trial. We have chosen to make it an algebraically decaying function of previously reported percepts per the previous trial in order to analyze the contribution to the results of possible *short-term* placebo effects or a *short-term* actual effects of ultrasound exposure.

After the application of the autoregression correlation term, which provides an initial reduction of the variance in the data, we then applied the rest of the GEE model to the data, which consisted of three mathematical expressions. The first was trial type: whether the trial consisted of sham or actual ultrasound exposure. The second expression was the number of ultrasound trials prior to the trial under consideration, independent of the current trial type. This mathematical expression sought to assay for a simple cumulative effect of ultrasound exposure, as it assumes the effects of a given exposure can last up until a given trial. The final expression was an interaction term, a function of the number of previous ultrasound trials as well as the current trial type. It seeks to identify a possible cumulative effect induced by previous ultrasound exposure on each of the actual vs. sham ultrasound trials.

The GEE model was implemented in the R language (R Core Team, 2018) using the software package geepack (Yan, 2002; Yan and Fine, 2004; Halekoh et al., 2006) the effect sizes were visualized using ggplot2 (Wickham, 2009).

Finally, we report all aggregate data in terms of mean ± standard error.

## Results

### Diagnostic Ultrasound Stimulation Elicited Visual Responses in Participants

Twenty of 21 participants observed phosphenes immediately after TMS exposure, thus providing functional confirmation that the target area corresponded to the visual cortex for those 20 participants, who then went on to the ultrasound phase of the study. Of the 10 participants subsequently exposed to sham plus actual ultrasound, seven reported percepts relative to baseline. Three of these participants did not. Of the 10 separate participants subsequently exposed to only sham ultrasound, seven also reported percepts relative to baseline while the remaining three did not.

For those exposed to sham plus actual ultrasound, percepts consisted most often of a variety of shapes such as rectangles, lines, and circles, or spots of color. Visual responses of a given participant were often identical or very similar throughout that participant’s experience. The colors of the percepts were also consistent across trials for a given participant. Gray was the most common color, followed by red and black. Three responsive participants did not assign a color to a sizable minority of their percepts. These observed percepts lasted most often between one and 10 s (4.6 ± 0.68 s), with fewer than 10% of them lasting less than a second or greater than 10 s. The onset of percepts was not tied to the immediate beginning of the ultrasound stimulation, but the effects almost always began and ended during the stimulation period with a mean onset of 7.8 ± 1.6 s. Table 1 summarizes the results.

**Table 1:** Ultrasound plus sham participant results: categories of participants, which participants were in each category, and a brief summary of the results from participants for ultrasound paradigm.

Participant group	Participants in group	Results
Responsive (7 participants)	Participant 1 Participant 2 Participant 3 Participant 7 Participant 8 Participant 10 Participant 11	4 total percepts, 2 “flashes” of the crosshair as the trial started. 2 images, both gray. 13 total percepts, blobs of gray color in an oblong or oval shape in 12/13 trials. 18 total percepts, 7 rectangles, 4 circles, 3 with lines. 3 total percepts, 2 trials where crosshair “flashed.” 13 total percepts, all were lines. During 3 trials participant reported percept before US stim had begun, during 2 trials participant reported percept approximately 40 s after the stimulation had ended. 2 total percepts, 2 lines that both lasted for 12 s. 6 total percepts, 4/6 were small dots
Non-Responsive (3 participants)	Participant 4 Participant 6 Participant 9	N/A
Non-Responsive to TMS and to US (1 participant)	Participant 5	N/A

For participants exposed to only sham ultrasound, their percepts were similar to those of participants exposed to each of the sham and actual ultrasound, consisting most often of a variety of shapes such as rectangles, lines, and circles, or spots of color. As above, the visual responses of a given participant were often identical or very similar throughout that participant’s experience. The colors of the percepts were also consistent across trials for a given participant. Gray was the most common color, followed by black, with one instance each of blue, green, and red. These observed percepts lasted between 0.16 and 8 s (3.0 ± 0.51 s). The onset of percepts was not tied to the immediate beginning of the stimulation period, but the effects always began and ended during the stimulation period with a mean onset of 8.21 ± 0.67 s. Table 2 summarizes the results.

**Table 2:** Sham only participant results: categories of participants, which participants were in each category, and a brief summary of the results from participants for control paradigm.

Participant group	Participants in group	Results
Responsive	Subject 1 Subject 4 Subject 6 Subject 7 Subject 8 Subject 9 Subject 10	17 total percepts, 11 of which were circles. 4 total percepts, 3 of which were lines 1 total percept, a blob of gray color 2 total percepts, one being crosshair shape and the other being a dot 7 total percepts, 5 being circles 8 total percepts, 7 beings spots of color 6 total percepts, 4 being spots of color
Non-responsive	Subject 2 Subject 3 Subject 5	N/A

Our different measures of observation times did not differ from one another in a statistically significant fashion between the sham-only and sham plus actual ultrasound cohorts.

### Report of Percept Per Trial

**Figure 7:**
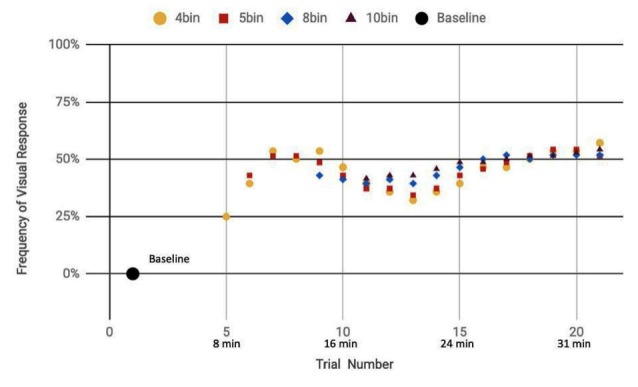
Reported visual response rate of participants exposed to a random mix of actual and sham DU vs. trial number and the corresponding time.

**Figure 8:**
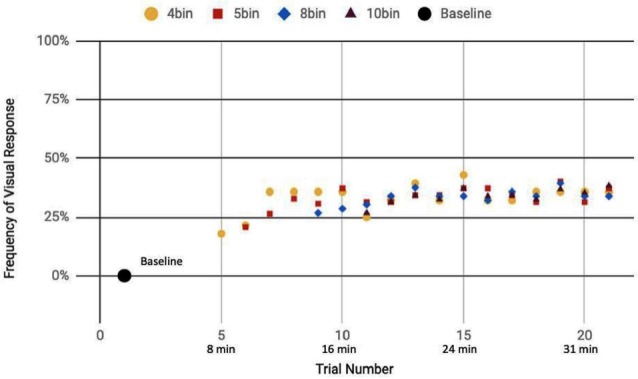
Reported visual response rate of participants exposed to a sham-only DU vs. trial number and the corresponding time.

Here, we analyze the 14 participants across both cohorts who reported observation of a percept relative to baseline, seven exposed to a mix of actual and sham ultrasound, another seven participants exposed to only sham ultrasound. Results of the sliding window analysis, when plotted with respect to trial number, indicated that for the seven “sham plus actual ultrasound” participants who experienced a percept, the number of trials for which they reported a percept first increased then plateaued by around 10 min over the remainder of the experiment to an average value of 53.7% ± 2.6% (Figure 7). Likewise, the group of participants who received an only sham ultrasound and observed a percept relative to baseline also experienced an initial increase in percept which then also plateaued at around 10 min and over the remainder of the experiment, but to a different average value, namely 36.3% ± 1.9% (Figure 8).

### Statistical Analysis

Statistical analysis of these two data sets (Table 3) showed that those exposed to a combination of sham and actual ultrasound (Figure 7) were, on average, more likely to report observing a percept than those exposed to purely sham DU (Figure 8). Since each group was blinded to their exposure conditions, these results support the primary objective of this study: participants more likely observe percepts when exposed to a mix of sham plus actual DU aimed towards their visual cortex than do those exposed to sham-only DU in the same fashion.

**Table 3:** Statistical analysis of sliding window data.

	4bin	5bin	8bin	10bin	Avg.
Kruskal–Wallis	*X*^2^: 12.2; *p* ≤ 0.0001	*X*^2^: 18; *p* ≤ 0.0001	*X*^2^: 18.3; *p* ≤ 0.0001	*X*^2^: 15.5; *p* ≤ 0.0001	*X*^2^: 65.4; *p* ≤ 0.0001
Two-sample Wilcoxon	*z*: 3.6; *p* ≤ 0.0004	*z*: 4.3; *p* ≤ 0.0001	*z*: 4.3; *p* ≤ 0.0001	*z*: 4; *p* ≤ 0.0001	*z*: 8.1; *p* ≤ 0.0001
R square, US + Sham	0.199	0.117	0.791	0.965	0.311
R square, Sham only	0.251	0.394	0.402	0.543	0.498

We have as a secondary hypothesis that ultrasound’s effects arise due to a combination of an immediate effect (that is, tied to a given exposure) and a cumulative effect (that is, tied to the net dose of ultrasound exposure). To answer this question, we applied the GEE model to all data from all participants who reported a percept when exposed to a mix of sham and actual ultrasound. The autoregression correlation term achieved an r squared value of 0.821. This means that 82.1% of the variance in the data arose due to a short-term effect ascribable to some combination of placebo and actual ultrasound influence on the participant’s likelihood of reporting a percept.

Further analysis with the GEE model showed that 17.25% of the total variance arose due to random variation (that is, phenomena not included in our statistical model) while our remaining model terms explain only 0.65% of the total variance—obviously of little use for explaining the variance in the data. Importantly, however, analysis of the remaining model terms allowed the identification of a statistically significant *net effect* in reported percepts over the course of the experiment attributable to a net, long-term effect of ultrasound exposure (Table 4).

**Table 4:** Outcomes of the GEE model after application of the AR-1 term.

Model term	The net change in reported percept by the end of the experiment	*p*-value
Number of previous ultrasound trials	+ 47.9 percentage points, independent of trial type	**0.014**
Interaction term	− 52.9 percentage points for sham trials relative to actual ultrasound trials	**0.027**
Trial type (Sham or Ultrasound)	+ 17.4 percentage point in sham trials relative to actual ultrasound trials	0.079

For example, analysis of the “number of previous ultrasound trials” term identified a significant long-term effect during a given trial and independent of trial type, due to previous, actual ultrasound exposure (*p* = 0.014): on average, participants exposed to a combination of sham and actual ultrasound reported a net increase of 47.9 percentage points in the likelihood that they would report a percept by the end of the experiment.

Also, analysis of the “interaction” term showed that the average likelihood of a participant reporting a percept after exposure to sham ultrasound decreased by 52.9 percentage points by the end of the experiment relative to those exposed to actual ultrasound (*p* = 0.027).

Finally, analysis of the “trial-type” term showed that the average likelihood of a participant reporting a percept after exposure to sham ultrasound tended to increase by the end of the experiment relative to those exposed to actual ultrasound without, however, rising to the level of statistical significance (*p* = 0.079).

## Discussion

### Diagnostic US Elicits Percepts

Our results show that a mix of actual and sham exposure of DU can increase by a factor of two the likelihood that a participant will report a change in their visual fields—their percepts—when applied directly over the portion of their visual cortex sensitive to TMS stimulation, relative to a sham exposed group.

The precepts observed by our participants differed significantly from those observed by Lee et al. (2016a), who reported new percepts after exposure of the visual cortex of participants to nDU while they had their eyes closed. Interestingly, those percepts appeared similar to TMS-induced phosphenes, in that the percepts appeared to spread across their field of vision and lasted for about a second. The percepts occurred concurrently and associated with the timing of stimulation intervals. In contrast, the percepts reported by our participants exposed to a combination of sham and actual DU trials frequently lasted longer (4.6 ± 0.68 s), appeared in and around the visual target rather than appearing diffusive in nature, and increased in likelihood due to subsequent exposures to actual ultrasound over a time span of 20 min.

Interestingly, our percentages of responsive and non-responsive subjects are similar to those reported by Lee et al. (2016a), discussed above. For Lee et al. (2016a), 11/19 or 58% of their participants responded to nDU, while 10.5% were partially responsive and the remaining 31.5% did not respond. In our study, 70% observed a percept while 30% did not.

The GEE analysis offers a deeper dive into these results. For example, the autoregression term shows that we can associate over 80% of the variance in observed percepts reported by our participants occurred due to either or both of placebo and actual effects of DU. Given that reports of percepts occurred, on average, twice as often for the sham plus actual ultrasound group as compared to the sham-only group (Figures 7, 8, respectively; Table 3), we provisionally ascribe half of this short-term effect experienced by our sham plus actual ultrasound cohort to actual influence by ultrasound on the function of their visual cortex during a given trial.

Analysis of the remaining less than 20% of the total *variance* in reported percept showed it arose predominantly due to phenomena not modeled by our GEE analysis. However, with that same analysis, we identified a *net effect* of DU exposure. Specifically, the “number of previous ultrasound trials” model term with the GEE analysis showed that over the course of the experiment, the accumulation of actual ultrasound exposures while a participant looked at the visual target resulted in a net increase in the likelihood of the participant reporting a percept associated with that target. This analysis along with details of the observations, further suggests that repeated exposures by DU made the visual cortex more responsive to stimulation by the visual target itself, rather than directly activating the visual cortex. To appreciate this assertion, note that the same number of participants reported percepts in each of our two cohorts (7 out of 10 in both). Also comparable were the length of time of the participant’s precepts as well as the kinds of percepts they experienced. Therefore, in contrast to the ability of TMS to generate phosphenes—the TMS percept that differs between sham and actual TMS—DU did not elicit *new* percepts. Instead, exposure to DU increased the likelihood that spontaneous visual percepts would arise in the subset of participants who reported them relative to those who never experienced actual DU. We hypothesize that participants have a natural tendency to spontaneously produce percepts during our experiment, perhaps as an effect of top-down attentional drives from higher-level cortical areas (Gilbert and Li, 2013; Zhang et al., 2014), and that DU increased the excitability of lower-level visual cortices, thus enhancing the effect.

Gibson et al. (2018) employed DU to achieve similar results by applying DU directly towards the human motor cortex: they found DU significantly increased cortical excitability inducible by subsequent TMS application to the same area. Our observations are also consistent with the discussion offered by Folloni et al. (2019) who used nDU to stimulate deep structures in the primate brain and observed changes downstream from their stimulation point. They said: “It is possible that (ultrasound) may act not simply by immediately inducing or reducing activity in neurons but by modulating their responsiveness to other neural inputs.” We agree.

Other researchers have also observed a lasting effect due to ultrasound stimulation of the brain. Yoo et al. (2011a) noticed altered visually evoked potentials for up to 35 min after nDU exposure of the somatosensory areas of anesthetized rats. The increased receptivity found by Gibson et al. (2018) discussed above lasted at least 6 min (but less than 11 min) after cessation of ultrasound exposure. Dallapiazza et al. (2018) reported decreased somatosensory evoked potentials for up to 10 min after targeting the thalamus of anesthetized pigs with nDU, which was the full extent of their measurement time. In a primate model, Folloni et al. (2019), discussed above, used nDU to create comparable effects that lasted more than an hour. Verhagen et al. (2019) demonstrated even longer-lasting effects from nDU stimulation. Using a 40 s nDU stimulation protocol applied to the supplementary motor area of macaques, they found regionally specific modulatory effects lasting up to 2 h after stimulation relative to controls.

A unique feature of our experiment relative to previous studies was the ultrasound frequency at which sensory effects occurred. An early study by Legon et al. (2014) noted that the optimal frequency for transcranial ultrasound was less than 0.65 MHz. Subsequent studies have substantially increased upwards the frequency range of ultrasound that can modulate brain function (Bobola et al., 2018). Gibson et al. (2018) used a DU transducer with a frequency range of 1–5 MHz and a central frequency of 2.32 MHz. In the present study, the transducer had a range of 6–13 MHz, and setting the device to penetration mode pushed this close to 6 MHz. The differences in DU frequency that successfully affected major cortical structures highlight a need for more research to improve our understanding of the optimal parameters for affecting brain function with DU.

### Limitations

Might our initial application of TMS sensitized the visual cortex of our test subjects to ultrasound stimulation throughout our experiment? This is at least a possibility, given that TMS can generate lasting modulatory effects (Ridding and Ziemann, 2010; Stagg and Nitsche, 2011). Nonetheless, we think this unlikely in our case for a number of reasons. First, our thresholding procedures adhered to the published guidelines that have consistently found no carry-over effects between single pulses separated by at least 5 s. In fact, the very existence of thresholding procedures such as the one employed in this study and previously used in other studies (Stocco et al., 2015; Losey et al., 2016; Jiang et al., 2019) depends on the documented lack of carry-over effects. Second, all of the established TMS protocols designed to have long-term effects (Ridding and Ziemann, 2010; Stagg and Nitsche, 2011) require many more pulses per second (e.g., 1 Hz or higher) and/or much longer exposure (e.g., 300 or 600 pulses). Third, those long-term behavioral effects dissipate within 10 min of their generation (Eisenegger et al., 2008), before the first application of sham or actual ultrasound in our experiment due to the time required to move the experiment from the TMS phase to the ultrasound phase. Finally, TMS stimulation occurred before we determined the baseline percept of each test subject, and we counted as percepts only those phenomena that differed from their baseline experience.

Our experiment lacked EEG, functional magnetic resonance imaging (fMRI) or other such independently measurable data to corroborate the visual effects reported by the participants. While our single-blinded experimental design with its two internal controls, results, and statistical analysis offer internally consistent evidence of ultrasound’s ability to alter the likelihood of our participants observing precepts, adding EEG or fMRI would have strengthened our results, which we will take into consideration for our next studies.

Our study represents, to our knowledge, the third (Hameroff et al., 2013; Gibson et al., 2018) report that *DU* can modulate brain function when applied through human skull. Taken together, these are surprising results given a low power and relatively high frequency of DU systems, whose emitted ultrasound should therefore minimally interact with the brain. These results are also intriguing, given the ubiquity of such systems, their ease of use, and currently low regulatory barriers to their use, hence their potential for brain research and for clinical applications. Might the effects we have observed have arisen, not due to a direct effect on the visual cortex, but instead due to an indirect mechanism, such as stimulation of peripheral nerves in the scalp by ultrasound and then translation of that signal into a visual percept? Such “cross-modal” sensory perception effects can certainly happen, including visual processing, most commonly studied for the case of simultaneous presentation of sound and a visual target (Shams and Kim, 2010). In our case, cross-modal sensory perception would arise most plausibly through generation by our DU of tingling or other sensations in the scalp while the participants viewed the visual target. None of our participants reported any such sensations, however. Nonetheless, had we EEG or fMRI data concurrent with the experiment we might have had in hand data that would support or refute this alternative hypothesis.

Our experimental design allowed for two controls—the sham-only group, and the use of a random distribution of sham exposures for the group exposed to actual DU. An alternative design could have used two study arms, one completely sham ultrasound, one completely actual ultrasound, with crossover of participants between the two arms. This would have ensured that we did not have an unusually sensitive group of participants in one group or another, at the expense of exposing them to more DU than we did by adding sham exposures in the “treatment” arm. Worth noting, DU has a strong safety profile (Blackmore et al., 2019); the use of nDU for neuromodulatory purposes has thus-far proven safe under most circumstances (Bobola et al., 2018; Blackmore et al., 2019) although its use and safety remains an area of active study (Blackmore et al., 2019). Another amendment to the experimental design would automate ultrasound delivery, thereby allowing us to implement a double-blinded study design rather than our single-blinded design.

Finally, we did not design our experiment to measure the time scale of the cumulative effect of the actual exposure of participants to DU, an area of study deserving more research and likely requiring at least a larger number of test subjects and a range of percentage of sham vs. actual ultrasound application.

## Conclusion

Ultrasound, with parameters typically outside FDA approved values for DU, can modulate brain function in a targeted and non-invasive way (Bobola et al., 2018; Blackmore et al., 2019). Our findings are consistent with the hypothesis that transcranially delivered *DU*, interleaved with a random distribution of sham DU, increased the tendency of our participants to report percepts during the course of the experiment by altering the receptivity of the visual cortex to stimulation by the visual target used in our study. This is consistent with the observations of Gibson et al. (2018), who found that transcranially delivered DU applied towards the motor cortex of healthy participants made that cortex more receptive to subsequent TMS stimulation as demonstrated by EMG measurements. We conclude that our observations along with those of Gibson et al. (2018) and Hameroff et al. (2013) make plausible the hypothesis that *DU* can have an effect on brain function. Future studies should identify the biophysical mechanism(s) and neural pathways by which DU, in our hands and others, has generated its observed effects on brain function. Given the pervasive use of DU throughout modern medicine, and given the ready availability of such systems due to their ubiquity and low regulatory barriers to their use, this phenomenon warrants further research, including into its potential clinical and therapeutic applications.

## Data Availability Statement

The datasets generated for this study are available on request to the corresponding author.

## Ethics Statement

The studies involving human participants were reviewed and approved by University of Washington’s Institutional Review Board. The patients/participants provided their written informed consent to participate in this study. Written informed consent was obtained from the individual(s) for the publication of any potentially identifiable images or data included in this article.

## Author Contributions

AS and PM conceived the presented idea. NS, JA, MB, AS, and PM designed the experiments. NS, JA, MS, and CB-C performed the experiments. ZB-C and MB performed the statistical analysis with guidance from PM. NS and PM wrote the majority of the article with meaningful contributions from the rest of the co-authors.

## Conflict of Interest

The authors declare that the research was conducted in the absence of any commercial or financial relationships that could be construed as a potential conflict of interest.

## Abbreviations

EEG: electroencephalography
GEE: generalized estimating equation
MRI: magnetic resonance imaging
DU: transcranially delivered diagnostic ultrasound
TMS: transcranial magnetic stimulation
nDU: transcranially delivered non-diagnostic ultrasound
US: ultrasound.
