# Supplementary material for: Repeated Application of Transcranial Diagnostic Ultrasound Towards the Visual Cortex Induced Illusory Visual Percepts in Healthy Participants
Source: Front Hum Neurosci. 2020 Mar 3;14:66. doi: 10.3389/fnhum.2020.00066 (PMC7062642; doi:10.3389/fnhum.2020.00066)
Supplement: Supplementary file 1 [file Data_Sheet_1.PDF]

“In our lab, we used TMS stimulation of the visual cortex to consistently produce phosphenes in participant’s visual fields, but we were unable to use TMS to reliably manipulate the shape, color, or location of produced phosphenes. However the ability to use TMS to produce phosphenes led to interest in seeing what stimulation of the visual cortex using ultrasound stimulation would produce, if anything. Since we are attempting to determine if diagnostic ultrasound has any capability of effecting the visual cortex, we are not sure whether the stimulation will produce anything.”

“We will use the TMS device, which stimulates the visual cortex by creating a magnetic field, coupled with a 3D neuro-navigation system to find an area of your visual cortex that when stimulated with TMS consistently produces phosphenes in your visual field. We will then target that area with the diagnostic ultrasound transducer, using the neuro-navigation system to keep the transducer within 3mm of the target area.”

“A single trial will consist of 2 seconds of increasing pink noise, 15 seconds of ultrasound stimulation with pink noise at a consistent level while you look at a gray screen with a white crosshair, 2 second of decreasing ultrasound, 45 seconds for drawing on the computer screen and verbally explaining any visual changes, then finally a 30 second survey displayed on the computer screen. The total experiment will consist of 21 trials, where the first trial is a baseline with no ultrasound stimulation.”

“If you see a visual change during a trial, press the spacebar once on the keyboard in front of you. This will start a stopwatch, allowing us to know when the onset of the visual change occurred. Once you no longer observe any visual changes, press the spacebar a second time to stop the stopwatch, allowing us to record how long the visual changes lasted. Once the stimulation phase is over, if you saw any visual changes you will have 45 seconds to draw them on the computer screen, and to verbally explain to us what you saw so we can record it. Then you will have 30 seconds to answer five questions about the visual change. If one of the questions does not relate to the visual changes you saw, or you saw no visual changes, there is a ‘N/A’ option for each question that you should select.”
